# Analytic solutions of the time-fractional Boiti-Leon-Manna-Pempinelli equation via novel transformation technique

**DOI:** 10.1038/s41598-025-00901-x

**Published:** 2025-05-20

**Authors:** Bushra Yasmeen, Khalil Ahmad, Ali Akgül, Qasem Al-Mdallal

**Affiliations:** 1 Department of Mathematics, Faculty of Basic and Applied Sciences, Air University, PAF Complex E-9, 44000 Islamabad, Pakistan; 2 Department of Electronics and Communication Engineering, Saveetha School of Engineering, SIMATS, Chennai, Tamilnadu India; 3 Art and Science Faculty, Department of Mathematics, Siirt University, 56100 Siirt, Turkey; 4 Department of Computer Engineering, Biruni University, Topkapi, 34010 Istanbul, Turkey; 5 Department of Mathematics, Mathematics Research Center, Near East University, Near East Boulevard, PC: 99138 Nicosia /Mersin 10, Turkey; 6 Department of Mathematical Sciences, UAE University, P.O. Box 17551, Al-Ain, UAE; 7 Applied Science Research Center, Applied Science Private University, Amman, 11937 Jordan

**Keywords:** Boiti-Leon-Manna-Pempinelli, Time-fractional Boiti-Leon-Manna-Pempinelli, Analytic solution, Euler’s second order linear ODE, Time-fractional derivative, Fractional calculus, New variable transformations, Applied mathematics, Materials science

## Abstract

This paper presents new analytical solutions for the time-fractional Boiti-Leon-Manna-Pempinelli (BLMP) equation, a crucial model for physical phenomena. Our approach yields novel wave solutions, whose propagation and dynamics are examined for diverse parameter values. The obtained solutions contain rational and natural logarithm functions. The graphical representations of the attained solutions are represented by plotted graphs with suitable parameters. The plotted graphs show different solitons and nonlinear wave solutions. The examination of these solutions involves a comprehensive analysis of their propagation and dynamics through analytic techniques. Our results with existing literature and found that our approach yields more accurate and efficient solutions. The novelty of these solutions is essential for understanding nonlinear behavior and natural phenomena. By developing analytical methods for nonlinear equations, this work advances our knowledge of complex systems. The results provide valuable insights into the equation’s behavior, shedding light on the underlying physical mechanisms. This research contributes to the development of analytical methods for nonlinear equations, fostering future research in the field. The findings are relevant to various areas of physics, including wave dynamics and nonlinear systems.

## Introduction

Natural phenomena are described by nonlinear partial differential equations (NPDEs) in a variety of fields, including biology and engineering. Particularly in engineering, nonlinear partial differential equations (NPDEs) have been used to simulate electromagnetic, hydrodynamic, and acoustic waves. The behavior of waves has been the focus of physics and engineering applications, thus scientists have been interested in finding answers to these problems for a long time^1^.

The idea of fractional derivatives has been successfully used in several scientific domains in recent years to mimic a variety of real-world occurrences. The history of a physical phenomena from its beginning condition to its current state is included in fractional order operators. As a result, model systems that explain the impact of memory effects frequently use fractional order operators in^2–4^ and^5^. Many scholars have been working to provide a new definition of the fractional derivative in recent years. As everyone knows, there are other ways to define fractional derivatives, including the Riemann-Liouville, modified Riemann-Liouville, and local fractional derivative senses. There are many other definitions of fractional derivatives in different ways. Recently,^6^ Khalil et al. have introduced a new, straightforward definition of the fractional derivative called the conformable fractional derivative with the limit operator^7^. We considered fractional derivatives because it addresses a significant gap in current research, has important implications and offers a unique opportunity to explore new ideas and approaches that can lead to innovative solutions and advancements.

### Definition 1:

**Caputo fractional derivative**

In^8^, the majority of Riemann liouville fractional derivative shortcomings were overcome by Caputo in 1967, when he made the most significant contributions to Fractioanl derivative

$$\begin{aligned} D_{x}^{\eta } f(x)=\frac{1}{A(n-\eta )}\frac{d^{n}}{dx^{n}}\int _{\alpha }^{x}(x-t)^{n-\eta -1}f(t)dt. \end{aligned}$$

### Definition 2:

**Grunwald-letnikov fractional derivative**

In^8^, Letnikov and Grunwald defined Fractional derivative of order $\eta$ in 1867, f(t) is given as,

$$\begin{aligned} f^{n}(x)= \lim \limits _{h \longrightarrow 0} \frac{1}{h^{n}} \sum _{j=0}^{\frac{t-\alpha }{h}} \frac{\Gamma (\eta +1)}{j!A(\eta -j+1)} \left( \begin{array}{c} \eta \\ j \end{array} \right) (-1)^{j}f(x-mh). \end{aligned}$$

### Definition 3:

**Conformable fractional derivative**

With the limit operator, the conformable fractional derivative is defined as follows:

$$ D_{t}^{r}(y(t)) = \lim \limits _{\sigma \longrightarrow 0} \frac{y(t+ \sigma t^{1-r})-y(t)}{\sigma }. $$

where $t >0$ , $r\epsilon (0,1]$ for a purpose $y = y(t):[0,\infty )\longrightarrow R$^9^. This definition is widely used in different fields, including physics, engineering, and mathematics, and has been shown to be effective in modeling complex phenomena. Other definitions, such as the Caputo and Grunwald-Letnikov definitions, also exist, but this definition is chosen for its simplicity and applicability to many problem.

The study of soliton and its application has been the subject of much research in the last several decades in^10–16^ and^17^. Solitons are accurate solutions of integrable equations in models of nonlinear systems of partial differential equations (NLSPDEs). After colliding with other solitons, they maintain their amplitude, speed, and form while moving at a steady pace. The nonlinearity and dispersive effects of a balanced medium lead to soliton formation.

The theory of fractional derivative is a very old theory, which dates back to a conversation on September 30, 1695, between L’Hopital and Leibniz concerning the definition of the operator $d^n /dx^n$ for $n=1/2$. Thus, as the time progresses, certain approaches have been given in the literature such as Riemann Lioville and that of Caputo. However, the development of theory of fractional calculus was due to Euler (1730), Lagrange (1849), Liouville and Riemann etc.^18^. Real or even complex order derivatives and integrals are deal with fractional derivatives. Through the use of polynomial fractional derivatives as

1 $$\begin{aligned} D_{t}^{\alpha }(t^{s})= \frac{\Gamma (s+1)}{\Gamma (s-\alpha +1)}t^{s-\alpha } \end{aligned}$$

### *Remark 1*

“All the fractional derivatives applying on polynomial have same results”.

### *Remark 2*

“The conformable fractional derivative is a powerful tool for modeling complex systems, offering a generalization of classical derivatives and enabling the accurate description of non-local and power-law behaviors. Its importance lies in its flexibility, simplification of complex problems and wide range of applications across various fields”.

## Problem statement

In paper^19^, (2+1)-dimensional Boiti-Leon-Manna-Pempinelli (BLMP) equation, which was derived by Boiti et al. (1986), is investigated as a class of KdV-type equation, reads as^20^

$$\begin{aligned} u_{yt} + u_{xxxy} - 3u_{xx}u_{y} - 3u_{x}u_{xy}=0, \end{aligned}$$

where $u = u(x, y, t)$ represents the physical quantity’s wave propagation, and the subscripts indicate partial differentiation with regard to the specified variables. The BLMP equation is a crucial model for incompressible fluids^21,22^.

In this paper, we consider following time-fractional BLMP equation as

2 $$\begin{aligned} D_{t}^{\alpha }u_{y} +u_{xxxy} -3u_{xx}u_{y} -3u_{x}u_{xy}=0. \end{aligned}$$

The results presented in this study provide a significant contribution to the existing literature on the topic. A thorough review of recent studies has been conducted to ensure the completeness and accuracy of the literature review. The BLMP equation has been extensively studied in recent years, and this work builds upon the findings of^21,22^.

The significance of this model holds in various areas of physics and mathematics. It plays a crucial role in soliton theory, as it admits soliton solutions that are essential in understanding nonlinear wave phenomena and has connections to other important equations. Physically, the BLMP equation is important because it describes various nonlinear phenomena in different fields, such as nonlinear optics, plasma physics and fluid dynamics. It provides a valuable tool for understanding and modeling complex nonlinear phenomena in various such fields.

The rest of this paper is as follows: we give steps of the transformation formulation in section “Problem statement”, then the choices of indices and solutions of transformations is given in section “Materials and methods”, and families are given in section “Discussion”. In final section “Conclusions”, we provide some conclusions based on the found wave solutions.

## Materials and methods

**Analytic solutions of BLMP by using new function transformations**

**Case A:** **(a) Formulation of Transformation**

We choose a new variable and function transformation as

3 $$\begin{aligned}  &   \xi =y^{m}z(t), \end{aligned}$$

4 $$\begin{aligned}  &   u =y^{n}A(t)f(\xi ) + ax^{k}B(t)y^{n}. \end{aligned}$$

Using chain rule, the partial derivative of *u* with respect to *y* can be found as

5 $$\begin{aligned} u_{y}=ny^{n-1}A(t)f(\xi )+my^{m+n-1}A(t)z(t)f'(\xi )+any^{n-1}x^{k}B(t) \end{aligned}$$

Using time-fractional derivative, given in (1), the Eq. (5) has the form

6 $$\begin{aligned}  &   D_{t}^{\alpha }(u_{y})=ny^{m+n-1}f'(\xi )D_{t}^{\alpha }z(t)A(t)+ my^{m+n-1}f'(\xi )D_{t}^{\alpha }z(t)A(t) \nonumber \\  &   \quad +my^{2m+n-1}A(t)z(t)D_{t}^{\alpha }z(t)f''(\xi ) \nonumber \\  &   \quad +ny^{n-1}f(\xi )D_{t}^{\alpha }A(t)+any^{n-1}x^{k}D_{t}^{\alpha }B(t) \end{aligned}$$

Again, differentiate (5), three times with respect to *x*, we get

7 $$\begin{aligned} u_{xxxy} = any^{n-1}k(k-1)(k-2)x^{k-3}B(t), \end{aligned}$$

Similarly, we need derivative of *u* in the following form

8 $$\begin{aligned} u_{xx}u_{y} =ak(k-1)x^{k-2}B(t)y^{n}\bigg ( ny^{n-1}A(t)f(\xi )+my^{m+n-1}A(t)z(t)f'(\xi )+any^{n-1}x^{k}B(t)\biggl ) , \end{aligned}$$

and

9 $$\begin{aligned} u_{x}u_{xy} = a^{2}k^{2}ny^{2n-1}x^{2k-2}B^{2}(t). \end{aligned}$$

Using (6–9), the PDE (2) can be expressed in the simplified form as:

10 $$\begin{aligned}  &   -3aA(t)B(t)z(t)x^{k-2}kf'(\xi )m(k-1)y^{2n-1+m} +y^{n+2m-1}A(t)f''(\xi )D_{t}^{\alpha }(z)mz(t) \nonumber \\  &   \quad -6ank\bigg (aB(t)(k-\frac{1}{2})x^{2k-2} +\frac{A(t)x^{k-2}f(\xi )(k-1)}{2}\bigg )B(t)y^{2n-1} \nonumber \\  &   \quad +\bigg (A(t)(n+m)D_{t}^{\alpha }(z) +z(t)D_{t}^{\alpha }(A)m\bigg )y^{n-1+m}f'(\xi ) \nonumber \\  &   \quad + y^{n-1}n\bigg (D_{t}^{\alpha }(A) f(\xi )+a\bigg [ B(t)k(k-1)(k-2)x^{k-3} +D_{t}^{\alpha }(B)x^{k}\bigg ] \bigg )=0. \end{aligned}$$

**Fig. 1:**
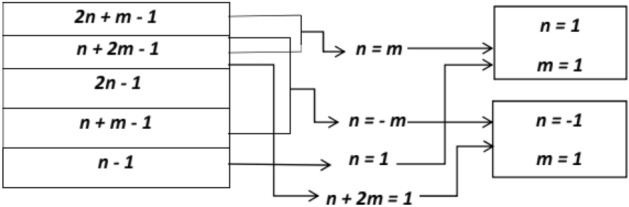
Selection criteria for indices.

Figure 1 shows selection criteria for indices.

**Case A:** **(b) Transformation optimization: Selecting ideal x and y indices**

**(i)**: m = 1, n = 1 and k = 0
**(ii)**: m = 1, n = 1 and a = 0
**(iii)**: m = 1, n = – 1 and k = 0

**Case B (i):** If we choose first case of above option i-e, **m = 1, n = 1** and **k = 0** in Eq. (10) which becomes

11 $$\begin{aligned} D_{t}^{\alpha }(A)f +yD_{t}^{\alpha }(A)f'z +2yAD_{t}^{\alpha }(z)f' +y^{2}AzD_{t}^{\alpha }(z)f'' +aB'=0 \end{aligned}$$

Dividing (11) by $$D_{t}^{\alpha }(A)$$

which becomes

12 $$\begin{aligned} f +yf'z +2y\frac{A}{D_{t}^{\alpha }(A)}D_{t}^{\alpha }(z)f' +y^{2}\frac{A}{D_{t}^{\alpha }(A)}zD_{t}^{\alpha }(z)f'' +a\frac{D_{t}^{\alpha }(B)}{D_{t}^{\alpha }(A)}=0 \end{aligned}$$

If we choose $$y\frac{A}{D_{t}^{\alpha }(A)}D_{t}^{\alpha }(z)= \frac{yz}{s}$$ implies $$s\frac{z}{D_{t}^{\alpha }(z)}=\frac{D_{t}^{\alpha }(A)}{A}$$, implies $$z^{s}=A$$ and again, we take $$y^{2}\frac{A}{D_{t}^{\alpha }(A)}zD_{t}^{\alpha }(z)= \frac{y^{2}z^{2}}{s},$$ we get $$z^{s}=A. $$

Since the last term in (12) cannot be change into $\xi$ due to missing *y* term, so it must be constant. So $\frac{D_{t}^{\alpha }(B)}{D_{t}^{\alpha }(A)}=1$, then $B=A.$

Finally we can choose $z=t^m$ , $A=t^n$ where $n=ms$, the Eqs. (3–5) reduce to

$$\begin{aligned}  &   \xi = yt^{m} \\  &   u= yt^{n}f(\xi ) + ayt^{n} \\  &   u_{y}=yt^{m+n}f'(\xi ) + ayt^{n} + ft^{n} \end{aligned}$$

Using Eq. (1) time-fractional derivative, we have

13 $$\begin{aligned}  &   D_{t}^{\alpha }u_{y}=\frac{\Gamma (m+n-1)}{\Gamma (m+n-\alpha +1)}yt^{m+n-\alpha }f'(\xi ) \nonumber \\  &   \quad +y^{2}t^{2m+n-\alpha }\frac{\Gamma (m+1)}{\Gamma (m-\alpha +1)}f''(\xi ) + \frac{\Gamma (n+1)}{\Gamma (n-\alpha +1)}t^{n- \alpha }(f+a) \end{aligned}$$

Since $$\xi =yt^{m}.$$

Using (13), the PDE (2) can be expressed in simplified as

14 $$\begin{aligned} \frac{\Gamma (n+1)}{\Gamma (n-\alpha +1)}(f(\xi )+a) + \frac{\Gamma (m+n-1)}{\Gamma (m+n-\alpha +1)}\xi f'(\xi ) +\xi ^{2}\frac{\Gamma (m+1)}{\Gamma (m-\alpha +1)}f''(\xi )=0 \end{aligned}$$

where $$b_{1}=\frac{\Gamma (m+1)}{\Gamma (m-\alpha +1)} $$, $$b_{2}=3\frac{\Gamma (m+n-1)}{\Gamma (m+n-\alpha +1)} $$ and $$b_{3}=\frac{\Gamma (n+1)}{\Gamma (n-\alpha +1)} .$$

It is an Euler second order linear and non-homogeneous ODE . If we choose $f(\xi )=\xi ^k$, $f'(\xi )=k\xi ^{k-1}$ and $f'' (\xi )= k(k-1)\xi ^{k-2}$ then the characteristic equation of homogeneous part is

$$\begin{aligned}  &   k(k-1)b_{1} + kb_{2} + b_{3}=0 \\  &   b_{1}k^{2}+ (b_{2}-b_{1})k +b_{3}=0 \\  &   k= \frac{b_{1}-b_{2} \pm \sqrt{(b_{2}-b_{1})^{2}-4b_{1}b{3}}}{2b_{1}} \end{aligned}$$

where $$ k_{1}= \frac{b_{1}-b_{2} + \sqrt{(b_{2}-b_{1})^{2}-4b_{1}b{3}}}{2b_{1}}$$ and $$k_{2}= \frac{b_{1}-b_{2} - \sqrt{(b_{2}-b_{1})^{2}-4b_{1}b{3}}}{2b_{1}}.$$

The exact solution is

$$\begin{aligned} f(\xi )=g(\xi )=c_{1}\xi ^{k_{1}} + c_{2}\xi ^{k_{2}} -a, \end{aligned}$$

The analytic solution of PDE (2) is

15 $$\begin{aligned}  &   u(t,y)= yt^{n} (c_{1}(yt^{m})^{k_{1}} + c_{2}(yt^{m})^{k_{2}}-a) +ayt^{n} \nonumber \\  &   u(t,y)= c_{1}y^{k_{1}+1}t^{mk_{1}+n} + c_{2}y^{k_{2}+1}t^{mk_{2}+n} \end{aligned}$$

**Families**

**Fig. 2:**
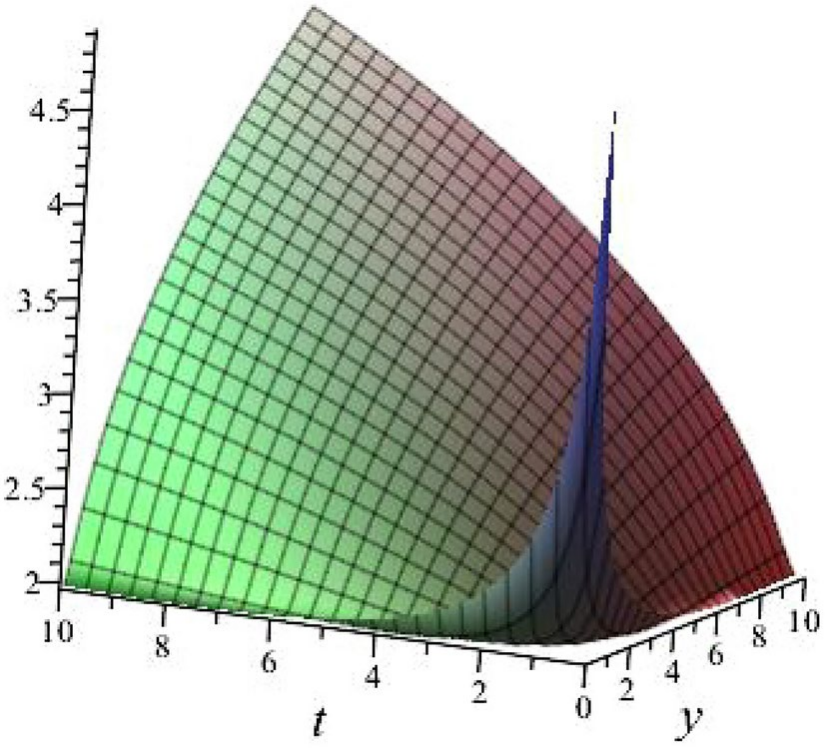
3D plot for solution of (16).

**(i)** **k = 1, m = 2, n = 1,** $$\alpha = 0.1$$, $$b_{1}=1.0397$$, $$b_{2}=3.2834$$, $$b_{3}=1.0397$$, $$k_{1}= -0.6738 $$, $$k_{2}=-1.4840 $$ is shown in Fig. 2

16 $$\begin{aligned} u(t,y)= c_{1}y^{\beta _{1}}t^{\gamma _{1}} + c_{2}y^{\beta _{2}}t^{\gamma _{2}}, \end{aligned}$$

where $$\beta _{1}= 0.3261$$, $$ \gamma _{1} = 0.3261$$, $$\beta _{2}= -0.4840$$ and $$ \gamma _{2} = -0.4840$$

**Fig. 3:**
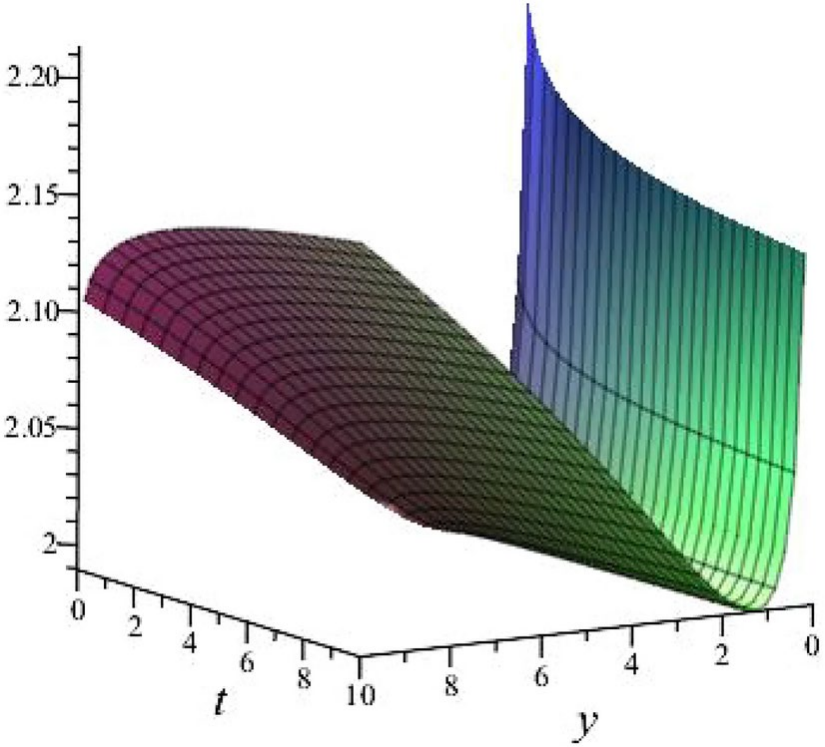
3D plot for solution of (17).

**(ii)** **k = 3, m = 0.1, n = 0.1,** $$\alpha = 0.1$$, $$b_{1}=0.9513$$, $$b_{2}=2.8953$$, $$b_{3}=0.9513$$, $$k_{1}= -0.8122 $$, $$k_{2}=-1.2312$$ is shown in Fig. 3

17 $$\begin{aligned} u(t,y)=c_{1}y^{\beta _{1}}t^{\gamma _{1}} + c_{2}y^{\beta _{2}}t^{\gamma _{2}}, \end{aligned}$$

where $$\beta _{1}= 0.1877$$, $$ \gamma _{1} = 0.01877$$, $$\beta _{2}=-0.2312 $$ and $$ \gamma _{2} =-0.02312 $$

**Case B (ii):** If we choose **n = 1, m = 1 and a = 0** in Eq. (10), which becomes

18 $$\begin{aligned} D_{t}^{\alpha }(A)f(\xi ) +yzD_{t}^{\alpha }(A)f'(\xi ) +2yAD_{t}^{\alpha }(z)f'(\xi ) +y^{2}AzD_{t}^{\alpha }(z)f''(\xi ) =0 \end{aligned}$$

Dividing (18) by $D_{t}^{\alpha }(A)$ which becomes

19 $$\begin{aligned} f + yzf' + 2yD_{t}^{\alpha }(z)\frac{A}{D_{t}^{\alpha }(A)}f' + y^{2}\frac{A}{D_{t}^{\alpha }(A)}zD_{t}^{\alpha }(z)f''=0 \end{aligned}$$

If we choose $z{D_{t}^{\alpha }(A)}= \frac{1}{2} A{D_{t}^{\alpha }(z)}$, implies $A= \sqrt{z}$

Finally we can choose $z=t^m$ , $A=t^{\frac{m}{2}}$, the Eqs. (3–5) reduce to

$$\begin{aligned}  &   \xi = yt^{m} \\  &   u= yt^{\frac{m}{2}}f(\xi ) \\  &   u_{y}=yt^{\frac{3m}{2}}f'(\xi ) + f(\xi )t^{\frac{m}{2}} \end{aligned}$$

Using Eq. (1) time-fractional derivative, we have

20 $$\begin{aligned}  &   D_{t}^{\alpha }u_{y}=\frac{\Gamma (\frac{3m}{2}+1)}{\Gamma (\frac{3m}{2}-\alpha +1)}yt^{\frac{3m}{2}-\alpha }f'(\xi ) \nonumber \\  &   \quad +y^{2}t^{\frac{5m}{2}-\alpha }\frac{\Gamma (m+1)}{\Gamma (m-\alpha +1)}f''(\xi ) + \frac{\Gamma (\frac{m}{2}+1)}{\Gamma (\frac{m}{2}-\alpha +1)}t^{n- \alpha }f(\xi ) + \frac{\Gamma (m+1)}{\Gamma (m-\alpha +1)}yt^{m+n-\alpha }f'(\xi ) \end{aligned}$$

Since $\xi =yt^{m}$. Using (20), the PDE (2) can be expressed in simplified as

21 $$\begin{aligned} \xi ^{2}\frac{\Gamma (m+1)}{\Gamma (m-\alpha +1)}f''(\xi ) + \bigg ( \frac{\Gamma (\frac{3m}{2}+1)}{\Gamma (\frac{3m}{2}-\alpha +1)} + \frac{\Gamma (m+1)}{\Gamma (m-\alpha +1)} \bigg )\xi f'(\xi ) + \frac{\Gamma (\frac{m}{2}+1)}{\Gamma (\frac{m}{2}-\alpha +1)}f(\xi ) =0 \end{aligned}$$

where $$b_{1}=\frac{\Gamma (m+1)}{\Gamma (m-\alpha +1)} $$, $$b_{2}= \frac{\Gamma (\frac{3m}{2}+1)}{\Gamma (\frac{3m}{2}-\alpha +1)} + \frac{\Gamma (m+1)}{\Gamma (m-\alpha +1)}$$ and $$b_{3}=\frac{\Gamma (\frac{m}{2}+1)}{\Gamma (\frac{m}{2}-\alpha +1)} $$

It is an Euler second order linear and homogeneous ODE . Since $f(\xi )=\xi ^l$, $f'(\xi )=l\xi ^{l-1}$ and $f'' (\xi )= l(l-1)\xi ^{l-2}$ then the characteristic equation of homogeneous part is

$$\begin{aligned}  &   l(l-1)b_{1} + lb_{2} + b_{3}=0 \\  &   b_{1}l^{2}+ (b_{2}-b_{1})l +b_{3}=0 \\  &   l= \frac{b_{1}-b_{2} \pm \sqrt{(b_{2}-b_{1})^{2}-4b_{1}b{3}}}{2b_{1}} \end{aligned}$$

where $$ l_{1}= \frac{b_{1}-b_{2} + \sqrt{(b_{2}-b_{1})^{2}-4b_{1}b{3}}}{2b_{1}}$$ and $$l_{2}= \frac{b_{1}-b_{2} - \sqrt{(b_{2}-b_{1})^{2}-4b_{1}b{3}}}{2b_{1}}$$

The exact solution is

$$\begin{aligned} f(\xi )=g(\xi )=c_{1}\xi ^{l_{1}} + c_{2}\xi ^{l_{2}}, \end{aligned}$$

The analytic solution of PDE (2) is

22 $$\begin{aligned}  &   u(t,y)= yt^{\frac{m}{2}} (c_{1}(yt^{m})^{l_{1}} + c_{2}(yt^{m})^{l_{2}}) \nonumber \\  &   \quad u(t,y)= c_{1}y^{l_{1}+1}t^{\frac{2ml_{1}+m}{2}} + c_{2}y^{l_{2}+1}t^{\frac{2ml_{2}+m}{2}} \end{aligned}$$

**Families**

**Fig. 4:**
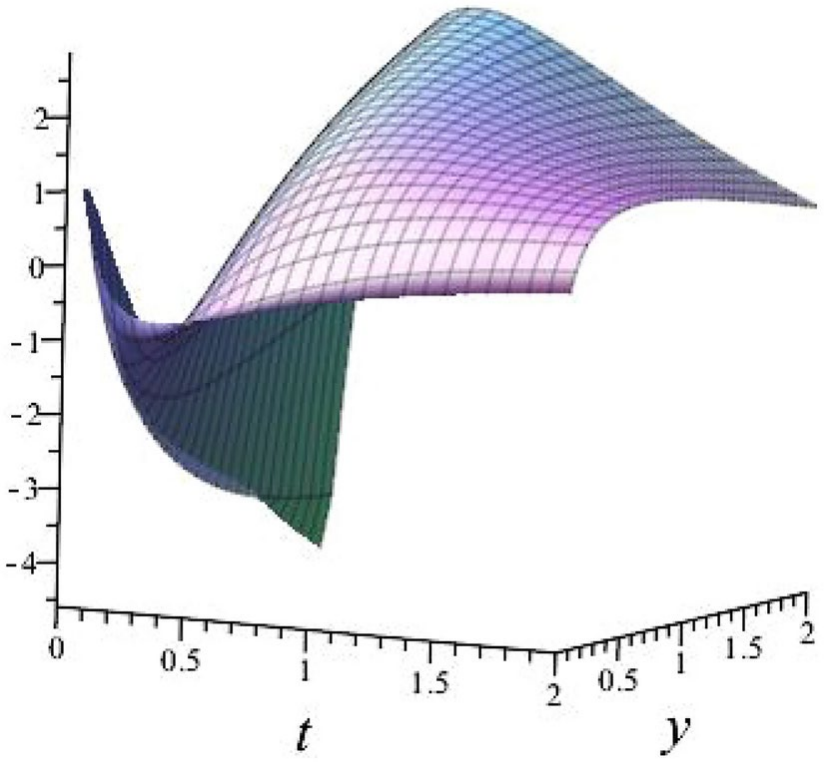
3D plot for solution of (23).

**(i)** **m = 2,** $$\alpha = 0.5$$, $$b_{1}=1.5054$$, $$b_{2}=3.3099$$, $$b_{3}=1.1283$$, $$l_{1}= -0.6000+ 0.62449$$, $$l_{2}=-0.6000- 0.62449 $$ shown in Fig. 4

23 $$\begin{aligned} u(t,y)= c_{1}y^{\beta _{1}}t^{\gamma _{1}} + c_{2}y^{\beta _{2}}t^{\gamma _{2}}, \end{aligned}$$

where $$\beta _{1}= 0.4 +0.6244$$, $$ \gamma _{1} = -0.2 +1.2489$$, $$\beta _{2}= 0.4 - 0.6244$$ and $$ \gamma _{2} = -0.2 - 1.2489$$

**Fig. 5:**
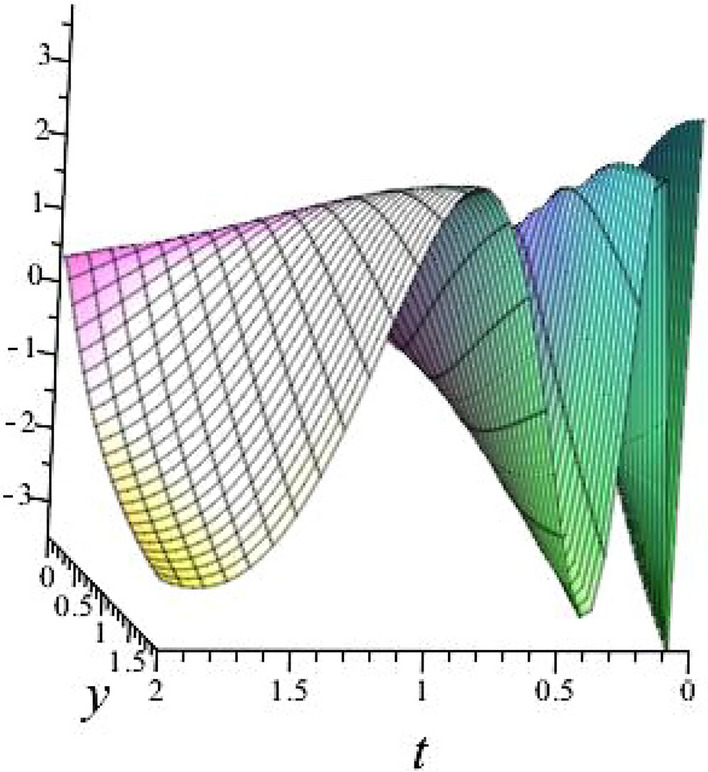
3D plot for solution of (24).

**(ii)** **m = 5, **$$\alpha = 0.1$$, $$b_{1}=1.1849$$, $$b_{2}=0.6942\sqrt{\pi } + 1.1849$$, $$b_{3}=0.6289\sqrt{\pi }$$, $$l_{1}= -0.8122 $$, $$l_{2}=-1.2312$$ shown in Fig. 5

24 $$\begin{aligned} u(t,y)= c_{1}y^{l_{1}+1}t^{\frac{2ml_{1}+m}{2}} + c_{2}y^{l_{2}+1}t^{\frac{2ml_{2}+m}{2}}, \end{aligned}$$

**Case B (iii):** If we choose **n = –1, m = 1 and k = 0** in Eq. (10) which becomes

$$\begin{aligned} Af''zD_{t}^{\alpha }(z) + \frac{D_{t}^{\alpha }(A)f'z}{y} - \frac{D_{t}^{\alpha }(A)f}{y^2} -\frac{aD_{t}^{\alpha }(B)}{y^2}=0 \end{aligned}$$

Multiply $y^2$, which becomes

25 $$\begin{aligned} y^{2}AzD_{t}^{\alpha }(z)f'' + D_{t}^{\alpha }(A)f'zy -D_{t}^{\alpha }(A)f -aD_{t}^{\alpha }(B)=0 \end{aligned}$$

Dividing (25) by $D_{t}^{\alpha }(A)$ which becomes

26 $$\begin{aligned} \frac{y^{2}zD_{t}^{\alpha }(z)Af''}{D_{t}^{\alpha }(A)} + f'yz -f-\frac{aD_{t}^{\alpha }(B)}{D_{t}^{\alpha }(A)}=0 \end{aligned}$$

If we choose $\frac{AzD_{t}^{\alpha }(z)}{D_{t}^{\alpha }(A)}= z^{2}$ implies $A=z$

Since the last term cannot be change into $\xi$ due to missing y term, so it must be constant in (25).

If $$\frac{D_{t}^{\alpha }(B)}{D_{t}^{\alpha }(A)}=1 $$ then $$A=B$$

Finally we choose $z=t^m$, $A=t^m$ where $m=ns$, the Eqs. (3–5) reduce to

$$\begin{aligned}  &   \xi = yt^{m} \\  &   u= \frac{t^{m}f(\xi )}{y} + \frac{at^{m}}{y} \\  &   u_{y}=\frac{t^{2m}f'(\xi )}{y} - \frac{t^{m}f(\xi )}{y^{2}} - \frac{at^{m}}{y^{2}} \end{aligned}$$

Using Eq. (1) time-fractional derivative, we have

27 $$\begin{aligned}  &   D_{t}^{\alpha }u_{y}= \frac{t^{2m}}{y} f'' y \frac{\Gamma (m+1)}{\Gamma (m-\alpha +)}t^{m-\alpha } + \frac{f'}{y} \frac{\Gamma (2m+1)}{\Gamma (2m-\alpha +)}t^{2m-\alpha } - \frac{t^{m}}{y^{2}} f'y \frac{\Gamma (m+1)}{\Gamma (m-\alpha +)}t^{m-\alpha } \nonumber \\  &   \quad - \frac{f}{y^{2}} \frac{\Gamma (m+1)}{\Gamma (m-\alpha +1)} t^{m-\alpha }- \frac{a}{y^{2}} \frac{\Gamma (m+1)}{\Gamma (m-\alpha +1)} t^{m-\alpha } \end{aligned}$$

Since $\xi =yt^{m}.$ Using (27), the PDE (2) can be expressed in simplified as

28 $$\begin{aligned} \xi ^{2}\frac{\Gamma (m+1)}{\Gamma (m-\alpha +1)}f''(\xi ) + \bigg (\frac{\Gamma (2m+1)}{\Gamma (2m-\alpha +1)}-\frac{\Gamma (m+1)}{\Gamma (m-\alpha +1)}\bigg )\xi f'(\xi ) - \frac{\Gamma (m+1)}{\Gamma (m-\alpha +1)}(f+a) =0 \end{aligned}$$

where $$b_{1}=\frac{\Gamma (m+1)}{\Gamma (m-\alpha +1)} $$, $$b_{2}= \frac{\Gamma (2m+1)}{\Gamma (2m-\alpha +1)}-\frac{\Gamma (m+1)}{\Gamma (m-\alpha +1)}$$ and $$b_{3}=-\frac{\Gamma (m+1)}{\Gamma (m-\alpha +1)} $$

It is an Euler second order linear and non-homogeneous ODE . If we choose $f(\xi )=\xi ^k$, $f'(\xi )=k\xi ^{k-1}$ and $f'' (\xi )= k(k-1)\xi ^{k-2}$ then the characteristic equation of homogeneous part is

$$\begin{aligned}  &   k(k-1)b_{1} + kb_{2} + b_{3}=0 \\  &   b_{1}k^{2}+ (b_{2}-b_{1})k +b_{3}=0 \\  &   k= \frac{b_{1}-b_{2} \pm \sqrt{(b_{2}-b_{1})^{2}-4b_{1}b{3}}}{2b_{1}} \end{aligned}$$

where $$ k_{1}= \frac{b_{1}-b_{2} + \sqrt{(b_{2}-b_{1})^{2}-4b_{1}b{3}}}{2b_{1}}$$ and $$k_{2}= \frac{b_{1}-b_{2} - \sqrt{(b_{2}-b_{1})^{2}-4b_{1}b{3}}}{2b_{1}}$$

The exact solution is

$$\begin{aligned} f(\xi )=g(\xi )=c_{1}\xi ^{k_{1}} + c_{2}\xi ^{k_{2}} -a, \end{aligned}$$

The analytic solution of PDE (2) is

29 $$\begin{aligned}  &   u(t,y)= \frac{t^{m}}{y} (c_{1}(yt^{m})^{k_{1}} + c_{2}(yt^{m})^{k_{2}}-a) +\frac{at^{m}}{y} \nonumber \\  &   u(t,y)= c_{1}y^{k_{1}-1} t^{mk_{1} +m}+ c_{2}y^{k_{2}-1}t^{mk_{2} +m} \end{aligned}$$

**Families**

**Fig. 6:**
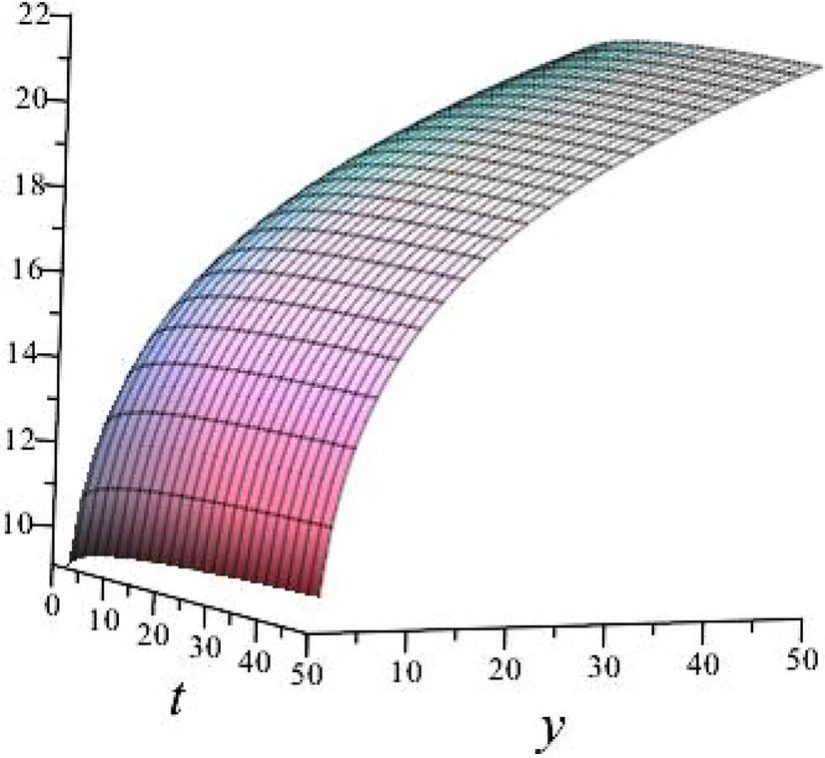
3D plot for solution of (30).

**(i)** **m = 2, n = 4,** $$\alpha = 0.1$$, $$b_{1}=0.9513$$, $$b_{2}=2.8953$$, $$b_{3}=0.9513$$, $$k_{1}= -0.8122 $$, $$k_{2}=-1.2312$$ shown in Fig. 6

30 $$\begin{aligned} u(t,y)=c_{1}y^{\beta _{1}}t^{\gamma _{1}} + c_{2}y^{\beta _{2}}t^{\gamma _{2}}, \end{aligned}$$

where $$\beta _{1}= 0.1877$$, $$ \gamma _{1} = 0.01877$$, $$\beta _{2}=-0.2312 $$ and $$ \gamma _{2} =-0.02312 $$

**Case C:** **(a) Formulation of Transformation**

We choose a function transformation

31 $$\begin{aligned}  &   \xi = t^{m}z(y) \end{aligned}$$

32 $$\begin{aligned}  &   u=t^{n}A(y)f(\xi ) \nonumber \\  &   u_{y}=A'(y)f(\xi )t^{n}+ A(y)f'(\xi )z'(y)t^{m+n} \end{aligned}$$

Using Eq. (1) time-fractional derivative, we have

33 $$\begin{aligned} D_{t}^{\alpha }u_y= \frac{\Gamma (m+1)}{\Gamma (m-\alpha +1)}t^{2m+n-\alpha }A(y)z(y)z'(y)f''(\xi ) + \frac{\Gamma (m+n+1)}{\Gamma (m+n-\alpha +1)}t^{m+n-\alpha }A(y)z'(y)f'(\xi ) \end{aligned}$$

Using (33), the PDE (2) can be expressed in simplified as;

34 $$\begin{aligned} \frac{\Gamma (n+1)}{\Gamma (n-\alpha +1)}A'f + \frac{\Gamma (m+1)}{\Gamma (m-\alpha +1)}A'f'zt^{m} + \frac{\Gamma (m+1)}{\Gamma (m-\alpha +1)}Azz'f''t^{2m} + \frac{\Gamma (m+n+1)}{\Gamma (m+n-\alpha +1)}Az'f't^{m}=0 \end{aligned}$$

**(b) Best Choices of Functions**

**(i)**: Since the coefficient term of $f''$ contains index $t^{2m}$, so it should be equal to $t^{2m}z^{2}$

Also, the coefficient of $f'$ contains $t^{m}$ term, it should be equal to $t^{m}z$

Solving $t^{2m}$ and $t^m$ simultaneously, we get $z=A$ and $A=y.$

**(ii)**: Since the coefficient term of $f''$ contains index $t^{2m}$, so it should be equal to $t^{2m}z^{2}$

Also, the coefficient of $f'$ contains $t^{m}$ term, it should be equal to $t^{m}z$

Solving $t^{2m}$ and $t^m$ simultaneously, we get $z=e^{y}$ and $A=1.$

**(iii)**: Since the coefficient term of $f''$ contains index $t^{2m}$, so it should be equal to $t^{2m}z^{2}$

Also, the coefficient of $f'$ contains $t^{m}$ term, it should be equal to $t^{m}z$

Solving $t^{2m}$ and $t^m$ simultaneously, we get $z=y^{-2}$ and $A=y.$

**(c) Explicit-form of transformations**

**(i)**: $$\xi =t^{m}y, u=t^{n}yf(\xi )$$
**(ii)**: $$\xi =t^{m}e^y, u=f(\xi )$$
**(iii)**: $$\xi =\frac{t^{m}}{y^{2}}, u=t^{n}yf(\xi )$$

**(d) Explanation**

**Case C (i):** $$\xi =t^{m}y, u=t^{n}yf(\xi )$$

Equation (34) becomes

35 $$\begin{aligned}  &   f \frac{\Gamma (n+1)}{\Gamma (n-\alpha +1)} + \xi \bigg ( \frac{\Gamma (m+1)}{\Gamma (m-\alpha +1)} + \frac{\Gamma (m+n+1)}{\Gamma (m+n-\alpha +1)} \bigg )f' + \xi ^{2}\frac{\Gamma (m+1)}{\Gamma (m-\alpha +1)} f''=0 \nonumber \\  &   \quad af + \xi bf' + \xi ^{2} df''=0 \end{aligned}$$

where $$a= \frac{\Gamma (n+1)}{\Gamma (n-\alpha +1)}$$, $$b=\frac{\Gamma (m+1)}{\Gamma (m-\alpha +1)} + \frac{\Gamma (m+n+1)}{\Gamma (m+n-\alpha +1)} $$ and $$d= \frac{\Gamma (m+1)}{\Gamma (m-\alpha +1)}.$$

It is easy to see that (35) is Euler second order linear ODE. If we choose $f=\xi ^{k}$, $f'=k\xi ^{k-1}$ and $f''=k(k-1)\xi ^{k-2}$, then the characteristic equation is

36 $$\begin{aligned} k(k-1)d + kb+a=0 \end{aligned}$$

The roots of equation (36) are $k=\frac{-(b-d)\pm \sqrt{(b-d)^{2}-4ad}}{2a}$

Hence the exact solution of ODE (35) is

$$\begin{aligned}  &   f_{1}(\xi )= c_{1}\xi ^{\frac{-(b-d)+\sqrt{(b-d)^{2}-4ad}}{2a}} \\  &   f_{2}(\xi )= c_{2}\xi ^{\frac{-(b-d)-\sqrt{(b-d)^{2}-4ad}}{2a}} \end{aligned}$$

The analytic solution of PDE (2) is;

37 $$\begin{aligned}  &   u_{1}(t,y) = c_{1}t^{n}y(t^{m}y)^{\frac{-(b-d)+\sqrt{(b-d)^{2}-4ad}}{2a}} \nonumber \\  &   \quad u_{2}(t,y) = c_{2}t^{n}y(t^{m}y)^{\frac{-(b-d)-\sqrt{(b-d)^{2}-4ad}}{2a}} \end{aligned}$$

where $$\alpha = b-d$$, $$\beta =\sqrt{(b-d)^{2}-4ad}$$ and $$\gamma =a$$

**Families**

**Fig. 7:**
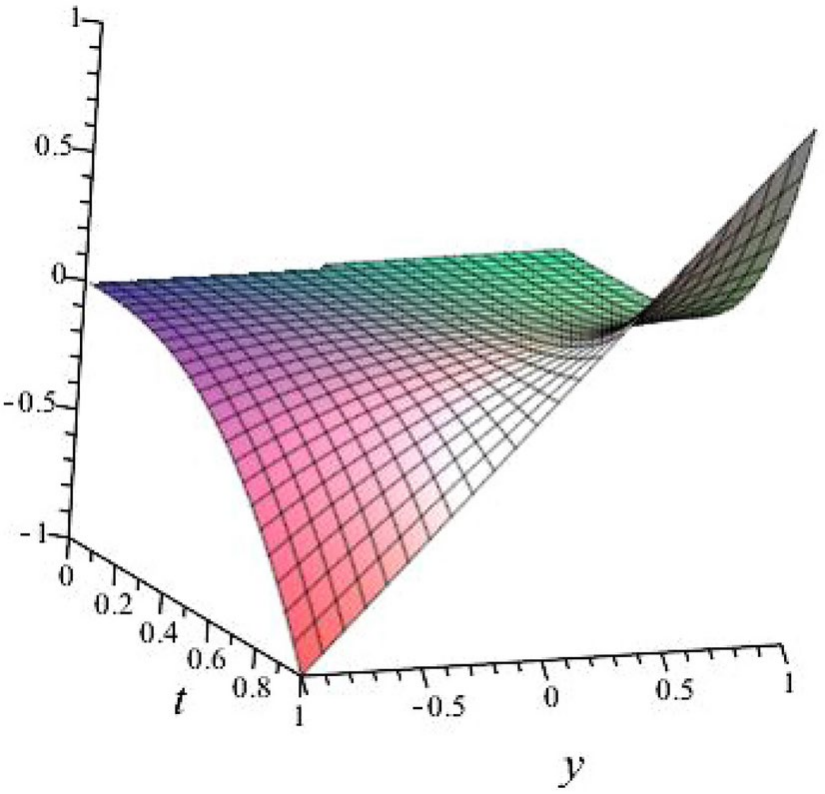
3D plot for solution of (38).

**(i)** $m=2$, $n=4$, $\alpha =-1$, $\beta =6$ and $\gamma =5$ shown in Fig. 7

38 $$\begin{aligned}  &   u_{1}= c_{1}t^{4}y(t^{2}y)^{\frac{7}{10}} \nonumber \\  &   \quad u_{2} = c_{2}t^{4}y(t^{2}y)^{\frac{7}{10}} \end{aligned}$$

**Fig. 8:**
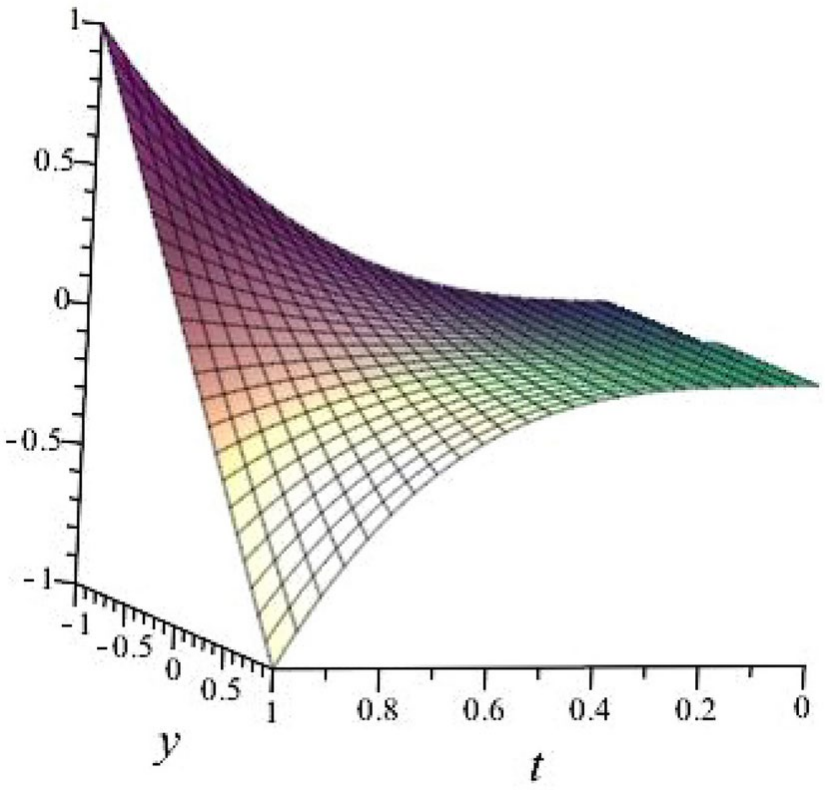
3D plot for solution of (39).

**(ii)** $m=-2$, $n=3$, $\alpha =-1$, $\beta =2$ and $\gamma =-5$ shown in Fig. 8

39 $$\begin{aligned}  &   u_{1}= c_{1}t^{3}y(t^{-2}y)^{\frac{-1}{5}} \nonumber \\  &   u_{2} = c_{2}t^{3}y(t^{-2}y)^{\frac{-1}{5}} \end{aligned}$$

**Case C (ii):** $$\xi = t^{m}e^{y}, u=f({\xi })$$

Equation (34) becomes

40 $$\begin{aligned}  &   \xi \frac{\Gamma (m+n+1)}{\Gamma (m+n-\alpha +1)} f' + \xi ^{2}\frac{\Gamma (m+1)}{\Gamma (m-\alpha +1)} f''=0 \nonumber \\  &   \quad \xi ^{2} f'' a + \xi f'b =0 \end{aligned}$$

where $$a= \frac{\Gamma (m+n+1)}{\Gamma (m+n-\alpha +1)} $$ and $$b=\frac{\Gamma (m+1)}{\Gamma (m-\alpha +1)}.$$

It is easy to see that (40) is Euler second order linear ODE. If we choose $f'=g$ and $f''=g'$, then the characteristic equation is

41 $$\begin{aligned}  &   \xi ^{2} g' a + \xi gb =0 \nonumber \\  &   \quad \frac{g'}{g}=-\frac{b}{\xi a} \end{aligned}$$

After integration, we get

$$\begin{aligned} g= \frac{c}{\xi ^{b/a}} \end{aligned}$$

Hence the exact solution is

$$\begin{aligned} f(\xi )= c_{1}ln\xi ^{b/a} +c_2 \end{aligned}$$

The analytic solution of PDE (2) is;

42 $$\begin{aligned} u(t,y) = c_{1}ln(t^{m} e^{y})^{b/a} +c_2 \end{aligned}$$

**Families**

**(i)** $$m=2$$, $$a=3$$ and $$b=-1$$

$$\begin{aligned} u = c_{1}ln(t^{m} e^{y})^{-1/3} +c_2 \end{aligned}$$

**(ii)** $$m=-2$$, $$a=2$$ and $$b=-1$$

$$\begin{aligned} u = c_{1}ln(t^{m} e^{y})^{-1/2} +c_2 \end{aligned}$$

**(iii)** $$m=2$$, $$a=2$$ and $$b=1$$

$$\begin{aligned} u = c_{1}ln(t^{m} e^{y})^{1/2} +c_2 \end{aligned}$$

**Case C (iii):** $$\xi =\frac{t^{m}}{y^{2}}, u=t^{n}yf(\xi )$$

Equation (34) becomes

43 $$\begin{aligned}  &   f \frac{\Gamma (n+1)}{\Gamma (n-\alpha +1)} + \xi \bigg ( \frac{\Gamma (m+1)}{\Gamma (m-\alpha +1)} -2 \frac{\Gamma (m+n+1)}{\Gamma (m+n-\alpha +1)} \bigg )f' + \xi ^{2}\bigg (-2\frac{\Gamma (m+1)}{\Gamma (m-\alpha +1)}\bigg ) f''=0 \nonumber \\  &   \quad af + \xi bf' + \xi ^{2} df''=0 \end{aligned}$$

where $$a= \frac{\Gamma (n+1)}{\Gamma (n-\alpha +1)}$$, $$b=\frac{\Gamma (m+1)}{\Gamma (m-\alpha +1)} -2 \frac{\Gamma (m+n+1)}{\Gamma (m+n-\alpha +1)} $$ and $$d=-2 \frac{\Gamma (m+1)}{\Gamma (m-\alpha +1)}.$$

It is easy to see that (43) is Euler second order linear ODE. If we choose $f=\xi ^{k}$, $f'=k\xi ^{k-1}$ and $f''=k(k-1)\xi ^{k-2}$, then the characteristic equation is

44 $$\begin{aligned} k(k-1)d + kb+a=0 \end{aligned}$$

The roots of equation (44) are $k=\frac{-(b-d)\pm \sqrt{(b-d)^{2}-4ad}}{2a}$

Hence the exact solution of ODE is

$$\begin{aligned}  &   f_{1}(\xi )= c_{1}\xi ^{\frac{-(b-d)+\sqrt{(b-d)^{2}-4ad}}{2a}} \\  &   f_{2}(\xi )= c_{2}\xi ^{\frac{-(b-d)-\sqrt{(b-d)^{2}-4ad}}{2a}} \end{aligned}$$

The analytic solution of PDE (2) is;

45 $$\begin{aligned}  &   u_{1}(t,y) = c_{1}t^{n}y(t^{m}y)^{\frac{-(b-d)+\sqrt{(b-d)^{2}-4ad}}{2a}} \nonumber \\  &   u_{2}(t,y) = c_{2}t^{n}y(t^{m}y)^{\frac{-(b-d)-\sqrt{(b-d)^{2}-4ad}}{2a}} \end{aligned}$$

where $$\alpha = b-d$$, $$\beta =\sqrt{(b-d)^{2}-4ad}$$ and $$\gamma =a$$

**Families**

**(i)** $$m=-3$$, $$n=2$$, $$\alpha =2$$, $$\beta =-1$$ and $$\gamma =2$$

$$\begin{aligned}  &   u_{1}= c_{1}t^{2}y(t^{-3}y)^{\frac{-3}{4}} \\  &   u_{2} = c_{1}t^{2}y(t^{-3}y)^{\frac{-3}{4}} \end{aligned}$$

**(ii)** $$m=-2$$, $$n=-3$$, $$\alpha =-1$$, $$\beta =-2$$ and $$\gamma =3$$

$$\begin{aligned}  &   u_{1}= c_{1}t^{-3}y(t^{-2}y)^{\frac{-1}{6}} \\  &   u_{2} = c_{2}t^{-3}y(t^{-2}y)^{\frac{-1}{6}} \end{aligned}$$

**(iii)** $$m=4$$, $$n=4$$, $$\alpha =4$$, $$\beta =-3$$ and $$\gamma =2$$

$$\begin{aligned}  &   u_{1}= c_{1}t^{4}y(t^{4}y)^{\frac{-7}{4}} \\  &   u_{2} = c_{1}t^{4}y(t^{4}y)^{\frac{-7}{4}} \end{aligned}$$

**Case D:** **Special case**

Now we choose mixed variables function transformation

46 $$\begin{aligned}  &   \xi =\frac{1}{(t-y)^m} \end{aligned}$$

47 $$\begin{aligned}  &   u=f(\xi ) \end{aligned}$$

Using chain rule, we have

$$\begin{aligned} u_{y}= \frac{m}{(t-y)^{m+1}}f'(\xi ) \end{aligned}$$

Using time-fractional derivative, we have

$$\begin{aligned} D_{t}^{\alpha }(u_y)=\frac{\Gamma (-m)}{\Gamma (-m-\alpha )}m(t-y)^{-m-1-\alpha }f'(\xi ) + \frac{\Gamma (-m+1)}{\Gamma (-m-\alpha +1)}m(t-y)^{-2m-1-\alpha }f''(\xi ) \end{aligned}$$

The PDE (2) can be expressed in the simplified form as:

48 $$\begin{aligned} \frac{\Gamma (-m)}{\Gamma (-m-\alpha )}f'(\xi ) + \frac{\Gamma (-m+1)}{\Gamma (-m-\alpha +1)}\xi f''(\xi )=0 \end{aligned}$$

For simplification, we take $f'=g$, $f''=g'$ then (48) becomes

49 $$\begin{aligned} \frac{\Gamma (-m)}{\Gamma (-m-\alpha )}g + \frac{\Gamma (-m+1)}{\Gamma (-m-\alpha +1)}\xi g'=0 \end{aligned}$$

It is easy to see that (49) is Euler second order linear ODE. If we choose $g(\xi )=\xi ^{k}$, $g'(\xi )=k\xi ^{k-1}$ then the characteristics equation of (49) is

50 $$\begin{aligned} k\frac{\Gamma (-m+1)}{\Gamma (-m-\alpha +1)} + \frac{\Gamma (-m)}{\Gamma (-m-\alpha )} =0 \end{aligned}$$

The exact solution is;

$$\begin{aligned} f'(\xi )=g(\xi )=c_{1}\xi ^{k}, \end{aligned}$$

where

51 $$\begin{aligned}  &   k= - \frac{\Gamma (-m)\Gamma (-m-\alpha +1)}{\Gamma (-m-\alpha )\Gamma (-m+1)} \nonumber \\  &   \quad f(\xi )= c_{1}\frac{\xi ^{k+1}}{k+1} + c_{2} \end{aligned}$$

The analytic solution of PDE (2) is;

52 $$\begin{aligned} u(t,y)=\frac{c_{1}}{k+1} (t-y)^{-mk-m}+ c_{2} \end{aligned}$$

**Families**

**Fig. 9:**
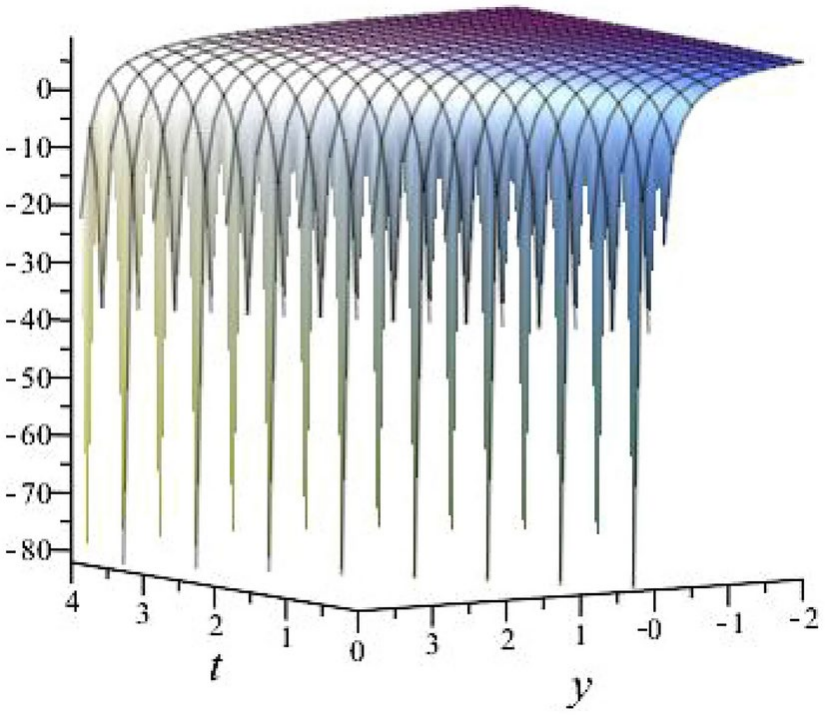
3D plot for solution of (53).

**(i)** $\alpha$** = 0.1, m = – 2** shown in Fig. 9

53 $$\begin{aligned} u= \frac{c_{1}}{0.05} (t-y)^{-0.95}+ c_{2} \end{aligned}$$

**Fig. 10:**
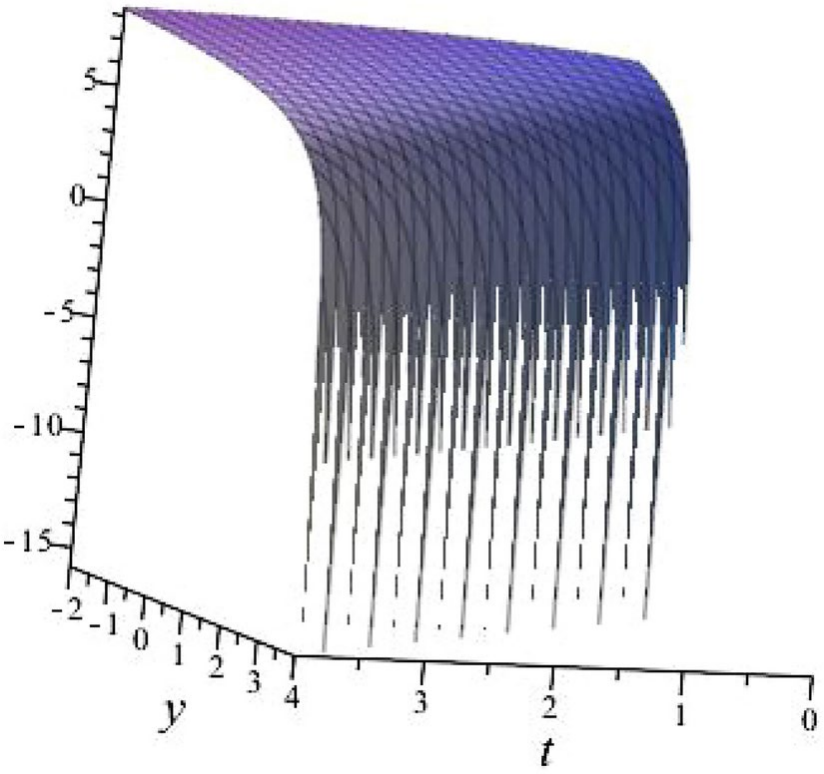
3D plot for solution of (54).

**(ii)** $\alpha =0.9$**, m = – 2** shown in Fig. 10

54 $$\begin{aligned} u= \frac{c_{1}}{0.45} (t-y)^{-0.55}+ c_{2} \end{aligned}$$

## Discussion

The discussion of this paper lies in the derivation of novel traveling wave solutions for the BLMP equation using time-fractional derivatives, which are distinct from existing solutions and offer new physical insights into the equation’s behavior, thereby extending the current literature and providing a promising direction for future research.

The methods used in this study provide rational and natural logarithm functions of the obtained solutions, which exhibits solitons and nonlinear wave solutions. The 3D plots provide a comprehensive view of the wave evolution in both space and time. Discuss how changes in height or curvature represent physical phenomena. The diversity of these soliton solutions highlights their importance in various fields, highlighting their potential impact on advancing our understanding and technical capabilities in nonlinear science and optics.

The above parameters can be changed to vary the shape and speed of the solitons and nonlinear waves, which will change the sensitivity and flexibility of the solutions that are produced. Time-space parameters can affect wave speed, spread, or frequency. Illustrate how different values change the figures and thus the system’s behavior, providing physical insights into how such parameters might be manipulated for desired outcomes in real-world application while fractional parameter explain the role of fractional derivatives in modeling memory effects or nonlocal interactions in the system.

## Conclusions

In this article, we have successfully derived novel traveling wave solutions for the Boiti-Leon-Manna-Pempinelli (BLMP) equation using time-fractional derivatives. Our results demonstrate a significant extension of existing solutions, showcasing diverse physical structures that reveal the complexity and richness of the equation’s behavior. The above figures shows that the interactions and interdependencies between three variables, highlighting their collective behavior and wave dynamics. The ease of implementation of our method makes it an attractive tool for future research, offering a promising avenue for exploring nonlinear wave phenomena. The ease of implementation of our paper is, highlighting its modular design, use of analytic methods and enhanced flexibility in handling complex wave phenomena. Our findings contribute meaningfully to the literature, providing new insights into the BLMP equation’s behavior and shedding light on its potential applications in various fields. The visual representations of our solutions vividly illustrate their physical significance, enriching our understanding of the underlying dynamics and highlighting the importance of this research. Overall, our work demonstrates the power of time-fractional derivatives in uncovering new aspects of nonlinear wave dynamics, opening up new avenues for future investigation and exploration.

Our findings show that the solutions involve rational and natural logarithm functions. The plotted graphs show different solitons and nonlinear wave solutions.

The results obtained are found to be more accurate and efficient in determining analytic solutions for time-fractional BLMP equations, demonstrating the effectiveness of the propose method. All of the findings presented in this research are fresh and original. These findings have potential applications in plasma physics and fluid dynamics. We concluded that our method is a robust tool for deriving analytical solutions to nonlinear evolution models.

Future research directions may include extending this methodology to address more intricate problems, such as systems of nonlinear PDEs including coupled time-fractional BLMP equations, which are crucial in modeling complex phenomena in physics, chaos theory and material sciences.

## Data Availability

The datasets used and analyzed during the current study are available in the manuscript. Any additional information or data required available from corresponding author upon reasonable request.
